# A roadmap to implementing machine learning in healthcare: from concept to practice

**DOI:** 10.3389/fdgth.2025.1462751

**Published:** 2025-01-20

**Authors:** Adam Paul Yan, Lin Lawrence Guo, Jiro Inoue, Santiago Eduardo Arciniegas, Emily Vettese, Agata Wolochacz, Nicole Crellin-Parsons, Brandon Purves, Steven Wallace, Azaz Patel, Medhat Roshdi, Karim Jessa, Bren Cardiff, Lillian Sung

**Affiliations:** ^1^ Division of Haematology/Oncology, The Hospital for Sick Children, Toronto, ON, Canada; ^2^ Program in Child Health Evaluative Sciences, The Hospital for Sick Children, Toronto, ON, Canada; ^3^ Information Management Technology, The Hospital for Sick Children, Toronto, ON, Canada; ^4^ Department of Emergency Medicine, The Hospital for Sick Children, Toronto, ON, Canada

**Keywords:** machine learning, clinical prediction models, implementation, clinical utilization, electronic health records

## Abstract

**Background:**

The adoption of machine learning (ML) has been slow within the healthcare setting. We launched Pediatric Real-world Evaluative Data sciences for Clinical Transformation (PREDICT) at a pediatric hospital. Its goal was to develop, deploy, evaluate and maintain clinical ML models to improve pediatric patient outcomes using electronic health records data.

**Objective:**

To provide examples from the PREDICT experience illustrating how common challenges with clinical ML deployment were addressed.

**Materials and methods:**

We present common challenges in developing and deploying models in healthcare related to the following: identify clinical scenarios, establish data infrastructure and utilization, create machine learning operations and integrate into clinical workflows.

**Results:**

We show examples of how these challenges were overcome and provide suggestions for pragmatic solutions while maintaining best practices.

**Discussion:**

These approaches will require refinement over time as the number of deployments and experience increase.

## Introduction

The application of machine learning (ML) is increasingly prevalent. However, ML adoption has been slower within the healthcare setting. Stages required to develop and deploy ML models in healthcare include the following: identify clinical scenarios, establish data infrastructure and utilization, create machine learning operations (MLOps) and integrate into clinical workflows. We launched Pediatric Real-world Evaluative Data sciences for Clinical Transformation (PREDICT) at The Hospital for Sick Children in 2023. The goal of PREDICT is to develop, deploy, evaluate and maintain clinical ML models to improve pediatric patient outcomes using electronic health records (EHR) data. Our objective was to provide examples from the PREDICT experience illustrating how common challenges with clinical ML deployment were addressed.

## Materials, methods and results

### Identify clinical scenario

One of the key challenges in applying ML in healthcare is identifying scenarios (or “use cases”) in which these approaches are useful and worthwhile. Careful identification of scenarios for deployment is important as healthcare resources are limited. Key factors to consider when determining if a clinical or operational problem is suitable for an ML solution include the problem being substantial, existence of a gap between current and desirable performance, and likelihood that ML with available data can result in improved performance (1). In a survey from two pediatric institutions, the most important attributes for prioritizing ML scenarios were risk stratification leading to differential actions and the clinical problem causing substantial morbidity or mortality (2).

Within PREDICT, we have provided additional considerations for scenario identification. To maximize the chances of a successful clinical deployment, we evaluate the healthcare team context and the clinical context. When evaluating the healthcare team context, all potential projects must be represented by a clinical champion who is willing to invest time and energy toward the project and a clinical steering group must be formed to ensure that multi-disciplinary stakeholders have awareness and input into the project. The clinical champion is also responsible for ensuring that the broader team is receptive to receiving ML predictions and using those predictions to drive patient care decisions. For the clinical context, we require that the targeted outcome (the label) be important, relatively common and measurable in the EHR. We also require an expectation that EHR data might be useful to predict the label. Finally, we require that knowing risk status would change clinical care in a fashion anticipated to favorably impact on patient outcomes or healthcare resources, and that the anticipated deployment environment has the change capacity to implement a new ML-based clinical workflow.

An example of a clinical scenario that met these criteria was vomiting prediction in pediatric oncology patients. Vomiting is considered one of the most dreaded side effects of cancer therapy and vomiting control rates are poor (3, 4). There are clinical practice guideline available (5–7) but yet, guideline-consistent care is uncommon (8). We proceeded with this project where the intervention for high-risk patients will include optimization of guideline-consistent antiemetic therapy.

Through our experience in soliciting and reviewing potential scenarios with clinical stakeholders, we have identified approaches that promote effective project exploration. Initially, we asked stakeholders to complete a standardized intake form that described the healthcare team and clinical contexts. However, we found clinical champions often did not have sufficient exposure to ML to complete the intake form in a fashion that allowed project evaluation. We have since shifted to a dynamic intake process where a clinical data scientist meets with potential end users to collaboratively complete the intake questionnaire together.

### Establish data infrastructure and utilization

Healthcare ML programs require clinical deployment environments where real-world data are accessible for developing and testing ML models at scale (9). However, real-world EHR data are complex, and data models evolve over time. Typically, ML development with EHR data occurs in trusted research environments, which often rely on custom, static extracts of data subsets that incur significant development costs (10), or on publicly available academic datasets (11). For PREDICT, we conceptualized and created the SickKids Enterprise-wide Data in Azure Repository (SEDAR) (12), a modular and robust approach to deliver foundational data that is re-usable across multiple ML projects. In addition to ML, SEDAR is currently being used to address institutional needs including administrative reporting, populating dashboards and enabling research and quality improvement projects.

SEDAR offers validated EHR data in a standardized and curated schema. This schema streamlines the EHR data into a unified structure of 18 tables organized by entities such as patients, visits, diagnoses, medications and laboratory results. These tables are relationally structured to support querying of longitudinal patient records and facilitate feature extraction for ML. Medical record number (MRN) and encounter identifiers (where applicable) enable linkage of patient- and encounter-specific data across tables while fields such as date-time, result, and description in tables such as laboratory results, diagnoses and medications enable precise temporal alignment of clinical events. While simple to navigate, the schema remains comprehensive and extensive, capturing diverse information about each patient's medical history in detail. This facilitates the extraction of thousands of clinical features for all patients across the institution, thus enabling the development of ML models capable of describing complex, longitudinal health patterns. Additionally, having centralized curation as a separate, intermediate step facilitates the management of changes in the source data model and their impact on downstream systems. Daily batch processes update the data model (with ongoing efforts to integrate live HL7 streams and FHIR APIs), and the data are loaded into centralized cloud storage, readily available for consumption.

With this structure, the SEDAR data schema is used to rapidly identify labels and efficiently create feature sets. Some cohorts or labels require in-depth clinical expertise and knowledge about the clinical workflow. For example, one project required identifying oncology patients with a cancer treatment plan. This effort required understanding the structure of treatment plans in the EHR and ensuring that this information is available prospectively. The cohort and label definitions can also introduce data leakage or bias into future models if they do not accurately reflect clinical workflows (13). For example, one project initially focused on identifying cardiac inpatients who will die or have a heart transplant. During data exploration, we realized that all heart transplant recipients were waitlisted prior to transplant, meaning that predicting heart transplant would not be a clinically meaningful endpoint since wait list status would already be known. Consequently, we modified the label to be death or waitlisted for transplant. Additional safeguards against data leakage include examining global explanations such as permutation feature importance (14) during model development to identify if a model relies on a feature that should not be available at the time of prediction; conducting ablation experiments to remove suspected proxy features that might indirectly reveal target outcome and examining their impact on performance; and running silent trials to evaluate the model on data and infrastructure that mirror deployment (see below).

Another issue to consider is algorithmic bias or fairness (15–17). SEDAR supports the evaluation of fairness by providing sex, age group, Epic non-English language flag, neighborhood income quintile and the four dimensions of the Canadian Index of Multiple Deprivation for each patient (race and ethnicity are not available in the SickKids EHR). Within PREDICT, we leverage this data to stratify model evaluations across all subpopulations of interest and analyze these results in collaboration with clinical champions. Fairness concerns may trigger explorations of use case design alternatives, including but not limited to the modeling stage (e.g., train different models or select different alert thresholds for different subpopulations). Satisfying all algorithmic fairness criteria is often not possible, making clinical champion inputs crucial to define the fairness goals for each use case. As part of our ongoing research efforts, we are exploring new fairness evaluation approaches based on recently proposed frameworks that may better inform decision making (18, 19).

Despite the advantages of centralized curation, the primary disadvantage is the resources and time required toward its initial creation. Also, SEDAR is currently tailored to a specific institution and may face challenges when scaled to multi-institutional settings. However, such challenges are common, even among sites using the same EHR. Data standards such as FHIR could enable greater interoperability, which would facilitate federated learning platforms (20).

### Create MLOps

**Figure 1:**
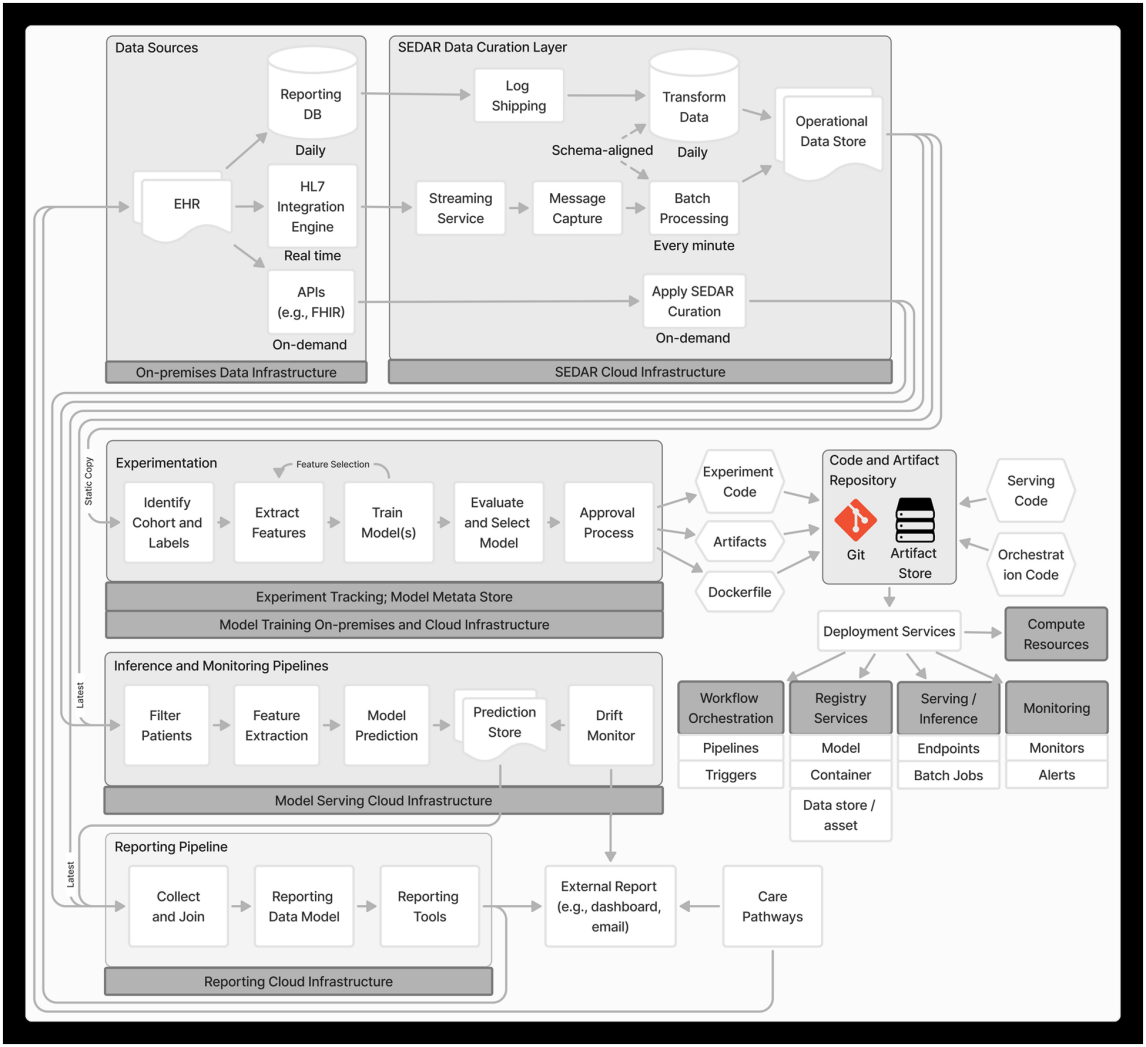
Machine learning operations architecture.

MLOps is a paradigm that integrates best practices across ML, software engineering and data engineering aimed at productionizing ML systems (21). Figure 1 depicts the end-to-end MLOps architecture in PREDICT, developed based on MLOps principles including automation and orchestration, modularity, versioning, reproducibility and monitoring.

#### Experimentation

For a given project, we implement a pipeline on a static set of identified cohort, label(s) and SEDAR data to enable reproducibility. This pipeline orchestrates modular, automated steps, including feature extraction, feature selection, model training, evaluation and model selection, with each step following a standard approach (see Supplementary Material for details). This orchestrated pipeline allows rapid experimentation with different features, model architectures, and model configurations to find the optimal set. Experiments are also tracked (22), storing metadata about the models and features.

An important consideration is that the pipeline must align with the model's anticipated use in production and the data generation process (23). For instance, vital signs often have multiple timestamps such as when the measurement was taken and a system-generated timestamp for when the data were entered into the EHR. Although the former more accurately reflects patient-specific events, the data are not available for inference until they have been entered, thus suggesting that data entry timestamp should be used.

#### Classification thresholds and approval

For classification tasks, a threshold is chosen to classify outcomes as positive or negative based on predicted probabilities. This process balances intervention downsides (such as personnel effort, resources, risks and alert burden) and the consequences of a missed outcome. Higher intervention downsides necessitate lower tolerance for false positives and thus, the need to maximize specificity and positive predictive value (PPV). Higher consequences of a missed outcome require lower tolerance for false negatives and thus, the need to maximize sensitivity and negative predictive value (NPV). Empirical data have demonstrated the potential for bias depending on the approach to threshold determination (24), highlighting the need for further evaluation in the context of ML.

For PREDICT use cases, operational performance data at multiple thresholds are presented to the clinical team, who can choose a threshold value based on both predictive performance and operational feasibility. Table 1 shows an example of data we present to the clinical team to facilitate threshold determination. Data include number of alerts, PPV and sensitivity for several thresholds derived from the retrospective model. Threshold selection approaches include maximizing true predictions (Youden's index) and different number needed to alert (NNA) values such as 2 and 3. This helps clinicians understand the potential impact of different thresholds. The threshold choice will be influenced by the intended intervention it triggers. For example, an alert from a model predicting mortality risk might recommend a palliative care consult. If specialist access is limited, a lower false positive rate is preferred. In this case, the team may select a low NNA such as NNA = 2, which would result in about 7 alerts/month in this example, but many false negatives. Alternatively, the primary team might apply additional clinical criteria such as assessing the patient's supportive care needs before deciding on a palliative care consult. This step wise approach permits a higher false positive rate since not all positive predictions lead to a consult, but it shifts the alert burden to the primary team while reducing specialist effort. In this case, the team may choose to maximize true predictions, which would result in about 49 alerts/month in this example, with a lower rate of false negatives. The number of alerts per month needs to be considered in the context of available resources. If the team does not have the ability to respond to 49 alerts/month, then the realized potential of the algorithm will not be met and an alternative threshold might be better suited (23). Involving diverse stakeholders in threshold discussions ensures balanced impacts on end users.

**Table 1:** Example of a hypothetical machine learning project with 3 different approaches to threshold determination^a^.

*N* = 930 (10 months)	Maximize True Predictions	NNA = 2	NNA = 3
Threshold	0.192	0.586	0.182
Total Alerts	494	72	520
True Positives	154	39	162
False Negatives	51	166	43
PPV	0.312	0.542	0.306
True Positives/Alerts	1/3	1/2	1/3
Sensitivity	0.75	0.19	0.79

NNA, number needed to alert; PPV, positive predictive value.

^a^ Thresholds were determined in the validation set and applied to the test set.

#### Model deployment

Upon approval, we commit the configurations, code and artifacts to Git repositories and secured cloud storage, which include components for feature extraction, patient selection, ML models, containers and services for serving, orchestrating and monitoring the pipeline. Software code for shared core components such as the featurizer and model training are separately versioned, packaged and released. We employ deployment services including continuous integration and continuous delivery pipelines to make each component available in the production environment. Where applicable, we run a suite of tests, including unit, integration and validation tests before deploying to production.

We leverage Azure ML's tools including compute resources, workflow orchestration, serving and registries. We register each component of the inference pipeline into the Azure ML Model Registry and Data Registry to automatically manage versioning, track data and model lineage, and facilitate integration with different Azure ML Pipelines and endpoints. Azure Container Registry is used to build, store and manage runtime container images. We set up scalable, on-demand computing resources using Azure ML managed compute clusters and configure Azure ML Pipelines for workflow orchestration. This includes defining the inference pipeline, setting up trigger schedules, and integrating monitoring and alerting systems.

Each project may require predictions to be delivered at different times and frequencies. In one project, predictions are required every morning before clinical rounds. Here, a time-based trigger initiates the inference pipeline once each morning. In another project, predictions are needed at a specific time before scheduled start of surgery. This requires an event-driven trigger that responds to real-time scheduling data to determine when the inference pipeline needs to run and creates a secondary time-based trigger to initiate the inference pipeline at the determined time.

An early technical consideration was whether to adopt a feature store system to centralize the storage of commonly used features and serve features at different latencies for experimentation vs. production. Although using a feature store aligns with best MLOps practices, we reasoned that applying custom configurations to a shared featurizer software for each project, instead of a centralized feature store, would allow us to quickly tailor feature extraction to the needs of each model. This flexibility is important because cohort definitions, best-performing features, and index times often differ across projects. We plan to re-evaluate our feature requirements in the future and reconsider the architecture. Additionally, we will consider other featurization approaches, such as using foundation models (25, 26), given their recent promise in performance (27), robustness (28–30) and efficiency (31).

#### Silent trial

The deployed model undergoes a silent trial where predictions are being generated in the production environment without being delivered to the end user. The duration of the silent trial is influenced by the outcome rate and the prediction window length. Once the clinical team decides that the model satisfies performance and utility criteria from data generated during the silent trial, the model is ready for clinical integration.

#### Continuous monitoring

Changes in patient population, healthcare practices or administration over time can lead to changes in the features or model predictions, ultimately causing model deterioration. Proactively preventing model deterioration is challenging (32), although some approaches are more robust than others (30, 33, 34). Knowledge of upcoming technical or clinical changes can aid in planning necessary adjustments to avoid disruptive shifts (35). However, it is anticipated that some models will have a limited life cycle due to irreparable model deterioration, availability of better models or approaches, operational or business requirement alterations and changes in clinical practice that make that model obsolete. Therefore, there is a need to monitor the ML system and adjust when needed.

Monitoring model performance should involve clinically meaningful metrics such as sensitivity and PPV. When labels are expensive (e.g., requires manual labeling) or there is a long prediction window (e.g., 6-month mortality risk), monitoring input data and model predictions against reference data (e.g., training data) using standard metrics can help detect potentially disruptive shifts. Criteria for model adjustments should focus on clinical impact (35). For example, the clinical team might decide that sensitivity or PPV lower than a certain threshold warrants model adjustments.

Within the PREDICT program, we monitor shift in features, model predictions and performance. Feature and prediction shift are measured using Jensen Shannon divergence, with development features and model predictions serving as the reference. Additionally, we monitor feature quality using percent missingness, percent out of range, and standard deviation. Model performance is assessed using both threshold-free (such as the area under the receiver operating characteristic curve) and threshold-based (such as PPV and sensitivity) metrics. Feature and prediction shift metrics are computed on a nightly basis over the previous month. Results are reported via Power BI dashboards.

Significant shifts in data, model predictions or performance may necessitate model re-calibration or re-training (36). However, it is important to consider whether the predictions lead to actions that may influence features or labels, as re-evaluation does not necessarily reflect performance in the absence of the intervention (37). For example, a model might lead to automatic ordering of a test. If the ordering of a test is the label, then re-evaluating the model does not reflect the underlying construct, which is whether a clinician would have ordered the test in the absence of a model. Similarly, if a model prediction leads to an intervention that reduces an undesirable outcome (e.g., clinical deterioration), the absence of the outcome might result from the successful intervention, not the initial “misprediction”. Addressing these challenges remains an open research question.

### Integrate into clinical workflows

#### Implementation considerations

ML implementation and supporting end users have been considered by multiple paradigms including change management, implementation sciences and quality improvement although some unique considerations will be required for ML. Within the PREDICT program, we engage with Clinical Informaticians who consider the end-to-end workflows and how potential electronic tools and solutions can be incorporated into clinical practice. This includes defining the clinical problem, designing the solution, validating and refining the solution, and evaluating the impact of the intervention.

Some model outputs may directly result in actions, such as re-ordering radiology queues. However, it is anticipated that many models will provide information to end users, who will then determine whether to act upon that prediction using their clinical judgement. If the intention of the model is for clinicians to act upon the prediction, implementation science considerations become critical to encourage behavioral change. Implementation interventions may include education, audit and feedback, and incentives, with additional in-person supports at initial deployment (38). Quantitative and qualitative evaluation will typically examine process measures (measuring steps that should be taken) and balancing measures (unintended negative consequences), and will identify facilitators and barriers to model uptake.

Other key considerations include the need for ongoing training and support for clinical staff following deployment, challenges maintaining end user engagement and the potential for resistance to change. Effective change management strategy is key toward sustained successful deployment (39–42).

#### Workflow and care pathways

As a component of implementation, the workflow of alerting recipients and articulating the intended interventions arising from the alert need to be determined. We have leveraged existing clinical decision support (CDS) frameworks such as the five rights of CDS (43) to guide the development of our approach for returning prediction results to clinicians. Model results may be provided to end users within the EHR via passive or interruptive alerts, or may be emailed to end users external to the EHR. For models that do not need immediate interventions, we have favored the return of predictions via email to a central person responsible for coordinating the dissemination of interventions that are required. For example, one project notifies pharmacists of which inpatients are at high risk of vomiting.

To specify the intended actions that should be taken based upon a positive prediction, we work with the end users to leverage their expertise, values and preferences. We then create a structured care pathway document to standardize how these actions are presented to users. The PREDICT process for care pathway creation and refinement generally follows a process we created to facilitate clinical practice guideline-consistent care (44, 45).

## Discussion

We have provided examples from the PREDICT experience illustrating how common challenges with clinical ML deployment were addressed. We have summarized our learnings as Table 2.

**Table 2:** Overview of the presented framework and key learnings.

Area	Key Learning
Identify clinical scenarios	Rather than asking stakeholders to complete a standardized intake form, we have since shifted to a dynamic intake process where a clinical data scientist meets with potential end users to collaboratively complete the intake questionnaire together.
Establish data infrastructure and utilization	We found that centralized curation and validation of electronic health records was an efficient approach that is re-usable across multiple machine learning projects.
Create MLOps	We found that standardization of model training and evaluation approach is efficient and re-usable across multiple machine learning projects. We also found that threshold selection based upon number needed to alert is effective to make decision making easier for clinicians.
Integrate into clinical workflows	Evaluation of facilitators and barriers is an important consideration to optimize implementation success. Broad stakeholder involvement and approval is required.

While this paper has reviewed approaches and challenges encountered within a pediatric setting, similar issues have been observed in adult settings. Such challenges include identifying appropriate clinical scenarios for ML (1), creation of clinical deployment environments (9), interoperability of EHR data (46), and implementation into clinical workflows (47). Consequently, most of these learning should be widely generalizable across different healthcare types.

A limitation of this paper is that we did not address the social and ethical implications of ML in healthcare. These are important issues that warrant fulsome debate and discussion among stakeholders, patients and families. Another limitation is that we do not report on the results of PREDICT clinical implementations. This type of reporting is an important future goal.

In conclusion, this paper provides practical recommendations for developing and deploying ML solutions in healthcare based upon the experiences at a single institution. These approaches will require refinement over time as the number of deployments and experience increase.

## Data Availability

The SickKids datasets presented in this article are not readily available because of the risks to patient privacy. Requests to access the dataset cannot be made.
