# Supplementary material for: A roadmap to implementing machine learning in healthcare: from concept to practice
Source: Front Digit Health. 2025 Jan 20;7:1462751. doi: 10.3389/fdgth.2025.1462751 (PMC11788154; doi:10.3389/fdgth.2025.1462751)
Supplement: Supplementary file 1 [file Datasheet1.pdf]

Supplementary Material: PREDICT Approach to Data Preprocessing, Training and Evaluating the Machine Learning Model

Extracting Features

We use a uniform approach to extracting features from the SickKids Enterprise-wide Data in Azure Repository (SEDAR), which we can then modify for specific use cases as required.(1, 2) We include demographic information (sex and age) and clinical observations over predefined time intervals prior to prediction time (0-1 days, 1-7 days and prior to 7 days). Clinical observations include categorical data (such as diagnosis codes) and continuous data (such as blood glucose levels). For categorical elements, we count occurrences within each interval. For continuous elements, we compute the mean, and for frequently observed measurements (those recorded more than twice on average for all patients), the minimum and maximum values within each interval. We exclude elements with fewer than 25 observations or those not observed in the last 90 days of the dataset.

Preprocessing steps include standardizing age and counts, imputing zeros for missing count values, and encoding measurement features into quintiles before one-hot encoding. This process results in a large, sparse feature matrix for each feature set. Each preprocessed feature set is then temporally split into training, validation and test sets in 70:15:15 ratio. All preprocessing steps are fit using the training set, for instance, determining quintile boundaries, and then applied to the validation and test sets.

Model Training and Evaluation

The training set is used to train L2-regularized logistic regression using Sci-kit Learn(3) and gradient boosting machines (GBMs) using LightGBM(4) and XGBoost(5). Hyperparameter selection is performed using 5-fold cross-validation, optimizing for area-under-the-receiver-operating-characteristic curve (AUROC). For feature selection, we recursively remove feature groups (derived from the same demographic information or clinical observation). These groups are ranked from lowest to highest by the maximum absolute value of coefficients for logistic regression or absolute gain for GBMs and removed in steps of 50%, 75%, 87.5%, 90%, 92.5% and 95%. At each step, we retrain and score the model five times, averaging the cross-validation AUROC. This process continues until the final step or until the cross-validation score decreases by more than 2% compared to the base model trained on all features.

The validation set is used to select model architecture (logistic regression, LightGBM or XGBoost) by optimizing AUROC. The test set is then used to express aggregate and subgroup-specific threshold-independent and threshold-dependent metrics. Threshold-independent metrics are AUROC, area-under-the-precision-recall curve and expected calibration error. Threshold dependent metrics are sensitivity, specificity, positive predictive value and negative predictive value.

REFERENCES

1. Rajkomar A, Oren E, Chen K, Dai AM, Hajaj N, Hardt M, et al. Scalable and accurate deep learning with electronic health records. NPJ Digit Med. 2018;1:18.
2. Reps JM, Schuemie MJ, Suchard MA, Ryan PB, Rijnbeek PR. Design and implementation of a standardized framework to generate and evaluate patient-level prediction models using observational healthcare data. J Am Med Inform Assoc. 2018;25(8):969-75.
3. Pedregosa F, Varoquaux G, Gramfort A, Michel V, Thirion B, Grisel O, et al. Scikit-learn: Machine Learning in Python. Journal of Machine Learning Research. 2011;12(85):2825-30.
4. Ke G, Meng Q, Finley T, Wang T, Chen W, Ma W, et al. LightGBM: A Highly Efficient Gradient Boosting Decision Tree. Advances in Neural Information Processing Systems 30 (NIPS 2017). 2017:3149–57
5. Chen T, Guestrin C. XGBoost: A Scalable Tree Boosting System. KDD '16: Proceedings of the 22nd ACM SIGKDD International Conference on Knowledge Discovery and Data Mining. 2016:785-94.
